# Clinical Research: A Review of Study Designs, Hypotheses, Errors, Sampling Types, Ethics, and Informed Consent

**DOI:** 10.7759/cureus.33374

**Published:** 2023-01-04

**Authors:** Addanki Purna singh, Sabitha Vadakedath, Venkataramana Kandi

**Affiliations:** 1 Physiology, Department of Biomedical Sciences, Saint James School of Medicine, The Quarter, AIA; 2 Biochemistry, Prathima Institute of Medical Sciences, Karimnagar, IND; 3 Clinical Microbiology, Prathima Institute of Medical Sciences, Karimnagar, IND

**Keywords:** ethical concerns, informed consent, sampling types, study hypothesis, research designs, clinical research, pandemics, microbial infectious diseases, emergence and re-emergence

## Abstract

Recently, we have been noticing an increase in the emergence and re-emergence of microbial infectious diseases. In the previous 100 years, there were several incidences of pandemics caused by different microbial species like the *influenza virus*, *human immunodeficiency virus* (HIV), *dengue virus*, *severe acute respiratory syndrome Coronavirus* (SARS-CoV), *middle east respiratory syndrome coronavirus* (MERS-CoV), and *SARS-CoV-2* that were responsible for severe morbidity and mortality among humans. Moreover, non-communicable diseases, including malignancies, diabetes, heart, liver, kidney, and lung diseases, have been on the rise. The medical fraternity, people, and governments all need to improve their preparedness to effectively tackle health emergencies. Clinical research, therefore, assumes increased significance in the current world and may potentially be applied to manage human health-related problems. In the current review, we describe the critical aspects of clinical research that include research designs, types of study hypotheses, errors, types of sampling, ethical concerns, and informed consent.

## Introduction and background

To conduct successful and credible research, scientists/researchers should understand the key elements of clinical research like neutrality (unbiased), reliability, validity, and generalizability. Moreover, results from clinical studies are applied in the real world to benefit human health. As a result, researchers must understand the various types of research designs [1]. Before choosing a research design, the researchers must work out the aims and objectives of the study, identify the study population, and address the ethical concerns associated with the clinical study. Another significant aspect of clinical studies is the research methodology and the statistical applications that are employed to process the data and draw conclusions. There are primarily two types of research designs: observational studies and experimental studies [2]. Observational studies do not involve any interventions and are therefore considered inferior to experimental designs. The experimental studies include the clinical trials that are carried out among a selected group of participants who are given a drug to assess its safety and efficacy in treating and managing the disease. However, in the absence of a study group, a single-case experimental design (SCED) was suggested as an alternative methodology that is equally reliable as a randomization study [3]. The single case study designs are called N-of-1 type clinical trials [4,5]. The N-of-1 study design is being increasingly applied in healthcare-related research. Experimental studies are complex and are generally performed by pharmaceutical industries as a part of research and development activities during the discovery of a therapeutic drug/device. Also, clinical trials are undertaken by individual researchers or a consortium. In a recent study, the researchers were cautioned about the consequences of a faulty research design [6]. It was noted that clinical studies on the effect of the gut microbiome and its relationship with the feed could potentially be influenced by the choice of the experimental design, controls, and comparison groups included in the study. Moreover, clinical studies can be affected by sampling errors and biases [7]. In the present review, we briefly discuss the types of clinical study designs, study hypotheses, sampling errors, and the ethical issues associated with clinical research.

## Review

Research design

**Figure 1:**
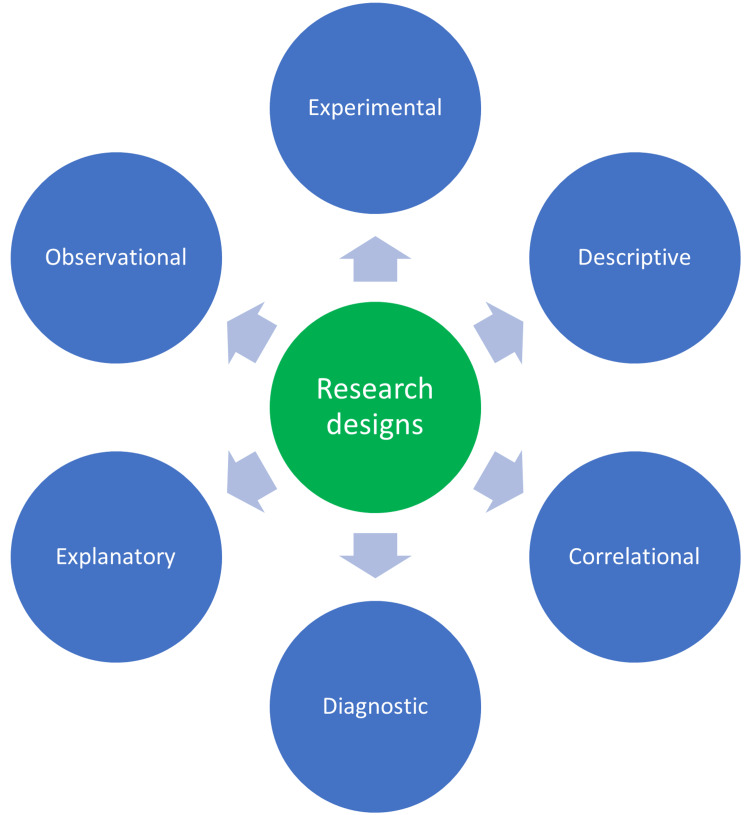
Types of research designs

A research design is a systematic elucidation of the whole research process that includes methods and techniques, starting from the planning of research, execution (data collection), analysis, and drawing a logical conclusion based on the results obtained. A research design is a framework developed by a research team to find an answer/solution to a problem. The research designs are of several types that include descriptive research, surveys, correlation type, experimental, review (systematic/literature), and meta-analysis. The choice of research design is determined by the type of research question that is opted for. Both the research design and the research question are interdependent. For every research question, a complementary/appropriate research design must have been chosen. The choice of research design influences the research credibility, reliability, and accuracy of the data collected. A well-defined research design would contain certain elements that include a specific purpose of the research, methods to be applied while collecting and analyzing the data, the research methodology used to interpret the collected data, research infrastructure, limitations, and most importantly, the time required to complete the research. The research design can broadly be categorized into two types: qualitative and quantitative designs. In a qualitative research method, the collected data are measured and evaluated using mathematical and statistical applications. Whereas in quantitative research, a larger sample size is selected, and the results derived from statistics can benefit society. The various types of research designs are shown in Figure 1 [8].

Types of research studies

**Figure 2:**
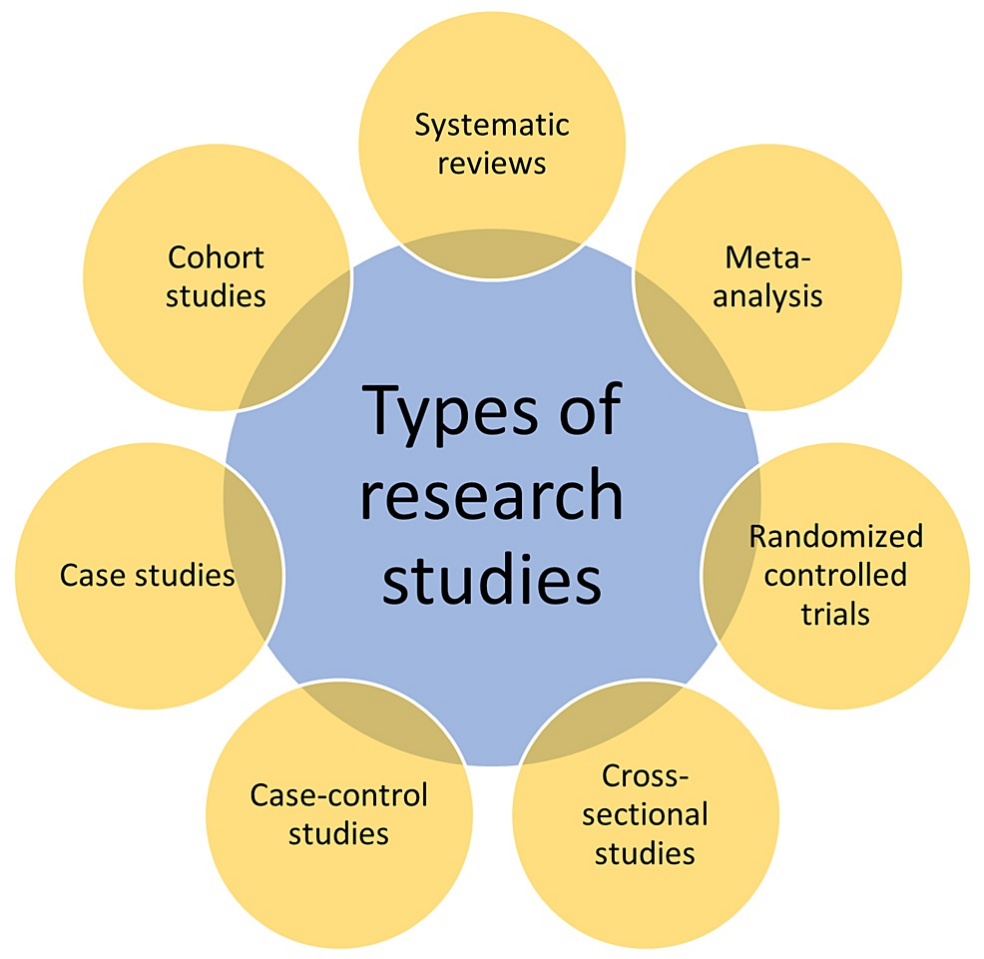
The types of research studies

There are various types of research study designs. The researcher who aims to take up the study determines the type of study design to choose among the available ones. The choice of study design depends on many factors that include but are not limited to the research question, the aim of the study, the available funds, manpower, and infrastructure, among others. The research study designs include systematic reviews, meta-analyses, randomized controlled trials, cross-sectional studies, case-control studies, cohort studies, case reports/studies, animal experiments, and other in vitro studies, as shown in Figure 2 [9].

Systematic Reviews

In these studies, the researcher makes an elaborate and up-to-date search of the available literature. By doing a systematic review of a selected topic, the researcher collects the data, analyses it, and critically evaluates it to evolve with impactful conclusions. Systematic reviews could equip healthcare professionals with more than adequate evidence with respect to the decisions to be taken during improved patient management that may include diagnosis, interventions, prognosis, and others [10]. A recent systematic research study evaluated the role of socioeconomic conditions on the knowledge of risk factors for stroke in the World Health Organization (WHO) European region. This study collected data from PubMed, Embase, Web of Science (WoS), and other sources and finally included 20 studies and 67,309 subjects. This study concluded that the high socioeconomic group had better knowledge of risk factors and warning signs of stroke and suggested improved public awareness programs to better address the issue [11].

Meta-Analysis

Meta-analysis is like a systematic review, but this type of research design uses quantitative tools that include statistical methods to draw conclusions. Such a research method is therefore considered both equal and superior to the original research studies. Both the systematic review and the meta-analyses follow a similar research process that includes the research question, preparation of a protocol, registration of the study, devising study methods using inclusion and exclusion criteria, an extensive literature survey, selection of studies, assessing the quality of the evidence, data collection, analysis, assessment of the evidence, and finally the interpretation/drawing the conclusions [12]. A recent research study, using a meta-analytical study design, evaluated the quality of life (QoL) among patients suffering from chronic pulmonary obstructive disease (COPD). This study used WoS to collect the studies, and STATA to analyze and interpret the data. The study concluded that non-therapeutic mental health and multidisciplinary approaches were used to improve QoL along with increased support from high-income countries to low and middle-income countries [13].

Cross-Sectional Studies

These studies undertake the observation of a select population group at a single point in time, wherein the subjects included in the studies are evaluated for exposure and outcome simultaneously. These are probably the most common types of studies undertaken by students pursuing postgraduation. A recent study evaluated the activities of thyroid hormones among the pre- and post-menopausal women attending a tertiary care teaching hospital. The results of this study demonstrated that there was no significant difference in the activities of thyroid hormones in the study groups [14].

Cohort Studies

Cohort studies use participant groups called cohorts, which are followed up for a certain period and assess the exposure to the outcome. They are used for epidemiological observations to improve public health. Although cohort studies are laborious, financially burdensome, and difficult to undertake as they require a large population group, such study designs are frequently used to conduct clinical studies and are only second to randomized control studies in terms of their significance [15]. Also, cohort studies can be undertaken both retrospectively and prospectively. A retrospective study assessed the effect of alcohol intake among human immunodeficiency virus (HIV)-infected persons under the national program of the United States of America (USA) for HIV care. This study, which included more than 30,000 HIV patients under the HIV care continuum program, revealed that excessive alcohol use among the participants affected HIV care, including treatment [16].

Case-Control Study

The case-control studies use a single point of observation among two population groups that are categorized based on the outcome. Those who had an outcome are termed as cases, and the ones who did not develop the disease are called control groups. This type of study design is easy to perform and is extensively undertaken as a part of medical research. Such studies are frequently used to assess the efficacy of vaccines among the population [17]. A previous study evaluated the activities of zinc among patients suffering from beta-thalassemia and compared it with the control group. This study concluded that the patients with beta-thalassemia are prone to hypozincaemia and had low concentrations of zinc as compared to the control group [18].

Case Studies

Such types of studies are especially important from the perspective of patient management. Although these studies are just observations of single or multiple cases, they may prove to be particularly important in the management of patients suffering from unusual diseases or patients presenting with unusual presentations of a common disease. Listeria is a bacterium that generally affects humans in the form of food poisoning and neonatal meningitis. Such an organism was reported to cause breast abscesses [19].

Randomized Control Trial

This is probably the most trusted research design that is frequently used to evaluate the efficacy of a novel pharmacological drug or a medical device. This type of study has a negligible bias, and the results obtained from such studies are considered accurate. The randomized controlled studies use two groups, wherein the treatment group receives the trial drug and the other group, called the placebo group, receives a blank drug that appears remarkably like the trial drug but without the pharmacological element. This can be a single-blind study (only the investigator knows who gets the trial drug and who is given a placebo) or a double-blind study (both the investigator and the study participant have no idea what is being given). A recent study (clinical trial registration number: NCT04308668) concluded that post-exposure prophylaxis with hydroxychloroquine does not protect against Coronavirus disease-19 (COVID-19) after a high and moderate risk exposure when the treatment was initiated within four days of potential exposure [20].

Factors that affect study designs

Among the different factors that affect a study's design is the recruitment of study participants. It is not yet clear as to what is the optimal method to increase participant participation in clinical studies. A previous study had identified that the language barrier and the long study intervals could potentially hamper the recruitment of subjects for clinical trials [21]. It was noted that patient recruitment for a new drug trial is more difficult than for a novel diagnostic study [22].

Reproducibility is an important factor that affects a research design. The study designs must be developed in such a way that they are replicable by others. Only those studies that can be re-done by others to generate the same/similar results are considered credible [23]. Choosing an appropriate study design to answer a research question is probably the most important factor that could affect the research result [24]. This can be addressed by clearly understanding various study designs and their applications before selecting a more relevant design.

Retention is another significant aspect of the study design. It is hard to hold the participants of a study until it is completed. Loss of follow-up among the study participants will influence the study results and the credibility of the study. Other factors that considerably influence the research design are the availability of a source of funding, the necessary infrastructure, and the skills of the investigators and clinical trial personnel.

Synthesizing a research question or a hypothesis

A research question is at the core of research and is the point from which a clinical study is initiated. It should be well-thought-out, clear, and concise, with an arguable element that requires the conduction of well-designed research to answer it. A research question should generally be a topic of curiosity in the researcher's mind, and he/she must be passionate enough about it to do all that is possible to answer it [25].

A research question must be generated/framed only after a preliminary literature search, choosing an appropriate topic, identifying the audience, self-questioning, and brainstorming for its clarity, feasibility, and reproducibility.

**Figure 3:**
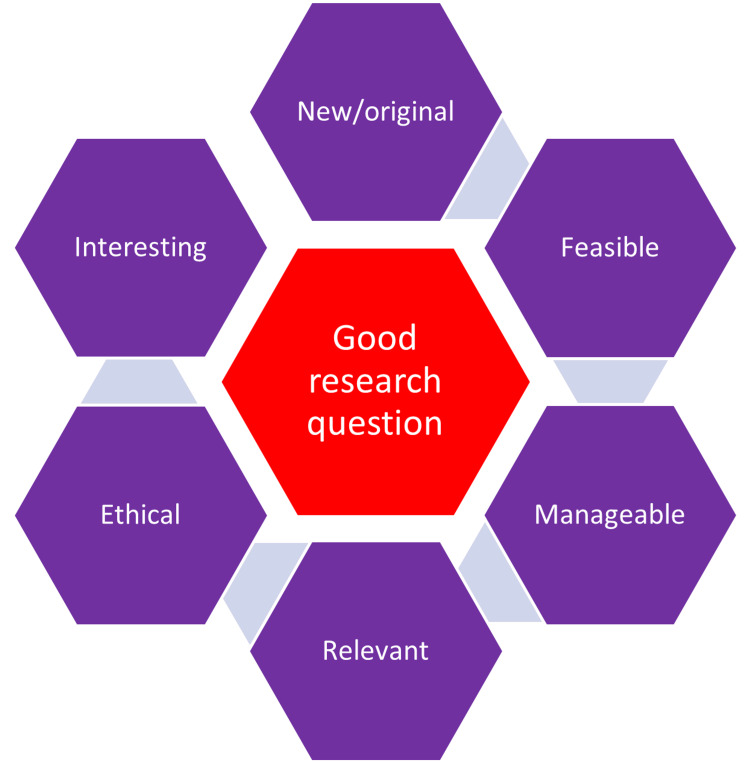
The elements of a good research question

A recent study suggested a stepwise process to frame the research question. The research question is developed to address a phenomenon, describe a case, establish a relationship for comparison, and identify causality, among others. A better research question is one that describes the statement of the problem, points out the study area, puts focus on the study aspects, and guides data collection, analysis, and interpretation. The aspects of a good research question are shown in Figure 3 [26].

Research questions may be framed to prove the existence of a phenomenon, describe and classify a condition, elaborate the composition of a disease condition, evaluate the relationship between variables, describe and compare disease conditions, establish causality, and compare the variables resulting in causality. Some examples of the research questions include: (i) Does the coronavirus mutate when it jumps from one organism to another?; (ii) What is the therapeutic efficacy of vitamin C and dexamethasone among patients infected with COVID-19?; (iii) Is there any relationship between COPD and the complications of COVID-19?; (iv) Is Remdesivir alone or in combination with vitamin supplements improve the outcome of COVID-19?; (v) Are males more prone to complications from COVID-19 than females?

The research hypothesis is remarkably like a research question except for the fact that in a hypothesis the researcher assumes either positively or negatively about a causality, relation, correlation, and association. An example of a research hypothesis: overweight and obesity are risk factors for cardiovascular disease.

Types of errors in hypothesis testing

An assumption or a preliminary observation made by the researcher about the potential outcome of research that is being envisaged may be called a hypothesis. There are different types of hypotheses, including simple hypotheses, complex hypotheses, empirical hypotheses, statistical hypotheses, null hypotheses, and alternative hypotheses. However, the null hypothesis (H0) and the alternative hypothesis (HA) are commonly practiced. The H0 is where the researcher assumes that there is no relation/causality/effect, and the HA is when the researcher believes/assumes that there is a relationship/effect [27,28].

Hypothesis testing is affected by two types of errors that include the type I error (α) and the type II error (β). The type I error (α) occurs when the investigator contradicts the null hypothesis despite it being true, which is considered a false positive error. The type II error (β) happens when the researcher considers/accepts the null hypothesis despite it being false, which is termed a false negative error [28,29].

The reasons for errors in the hypothesis testing may be due to bias and other causes. Therefore, the researchers set the standards for studies to rule out errors. A 5% deviation (α=0.05; range: 0.01-0.10) in the case of a type I error and up to a 20% probability (β=0.20; range: 0.05-0.20) for type II errors are generally accepted [28,29]. The features of a reasonable hypothesis include simplicity and specificity, and the hypothesis is generally determined by the researcher before the initiation of the study and during the preparation of the study proposal/protocol [28,29].

The applications of hypothesis testing

A hypothesis is tested by assessing the samples, where appropriate statistics are applied to the collected data and an inference is drawn from it. It was noted that a hypothesis can be made based on the observations of physicians using anatomical characteristics and other physiological attributes [28,30]. The hypothesis may also be tested by employing proper statistical techniques. Hypothesis testing is carried out on the sample data to affirm the null hypothesis or otherwise.

An investigator needs to believe the null hypothesis or accept that the alternate hypothesis is true based on the data collected from the samples. Interestingly, most of the time, a study that is carried out has only a 50% chance of either the null hypothesis or the alternative hypothesis coming true [28,31].

Hypothesis testing is a step-by-step strategy that is initiated by the assumption and followed by the measures applied to interpret the results, analysis, and conclusion. The margin of error and the level of significance (95% free of type I error and 80% free of type II error) are initially fixed. This enables the chance for the study results to be reproduced by other researchers [32].

Ethics in health research

Ethical concerns are an important aspect of civilized societies. Moreover, ethics in medical research and practice assumes increased significance as most health-related research is undertaken to find a cure or discover a medical device/diagnostic tool that can either diagnose or cure the disease. Because such research involves human participants, and due to the fact that people approach doctors to find cures for their diseased condition, ethics, and ethical concerns take center stage in public health-related clinical/medical practice and research.

The local and international authorities like the Drugs Controller General of India (DCGI), and the Food and Drug Administration (FDA) make sure that health-related research is carried out following all ethical concerns and good clinical practice (GCP) guidelines. The ethics guidelines are prescribed by both national and international bodies like the Indian Council of Medical Research (ICMR) and the World Medical Association (WMA) Declaration of Helsinki guidelines for ethical principles for medical research involving human subjects [33].

**Figure 4:**
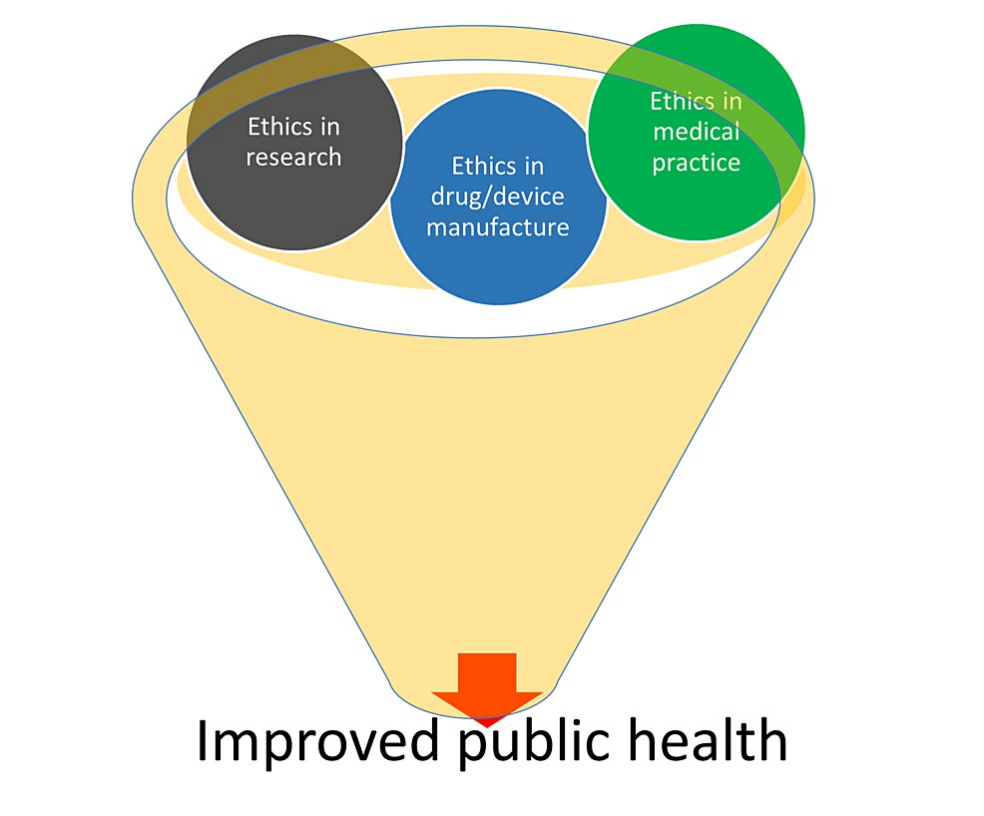
Ethics in research, practice, and manufacture

Ethical conduct is more significant during clinical practice, medical education, and research. It is recommended that medical practitioners embark on self-regulation of the medical profession. Becoming proactive in terms of ethical practices will enhance the social image of a medical practitioner/researcher. Moreover, such behavior will allow people to comprehend that this profession is not for trade/money but for the benefit of the patients and the public at large. Administrations should promote ethical practitioners and penalize unethical practitioners and clinical research organizations. It is suggested that the medical curriculum should incorporate ethics as a module and ethics-related training must be delivered to all medical personnel. It should be noted that a tiny seed grows into an exceptionally gigantic tree if adequately watered and taken care of [33]. It is therefore inevitable to address the ethical concerns in medical education, research and practice to make more promising medical practitioners and acceptable medical educators and researchers as shown in Figure 4.

Sampling in health research

**Figure 5:**
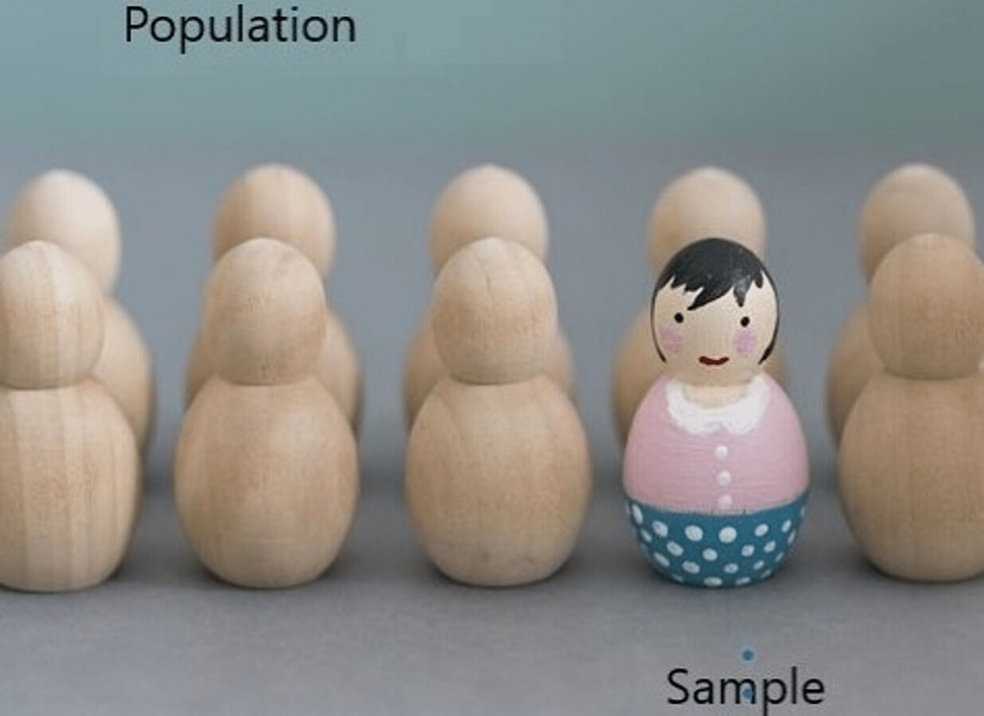
Representation of a sample from the population

Sampling is the procedure of picking a precise number of individuals from a defined group to accomplish a research study. This sample is a true representative subset of individuals who potentially share the same characteristics as a large population, and the results of the research can be generalized [34,35]. Sampling is a prerogative because it is almost impossible to include all the individuals who want to partake in a research investigation. A sample identified from a representative population can be depicted in Figure 5.

**Figure 6:**
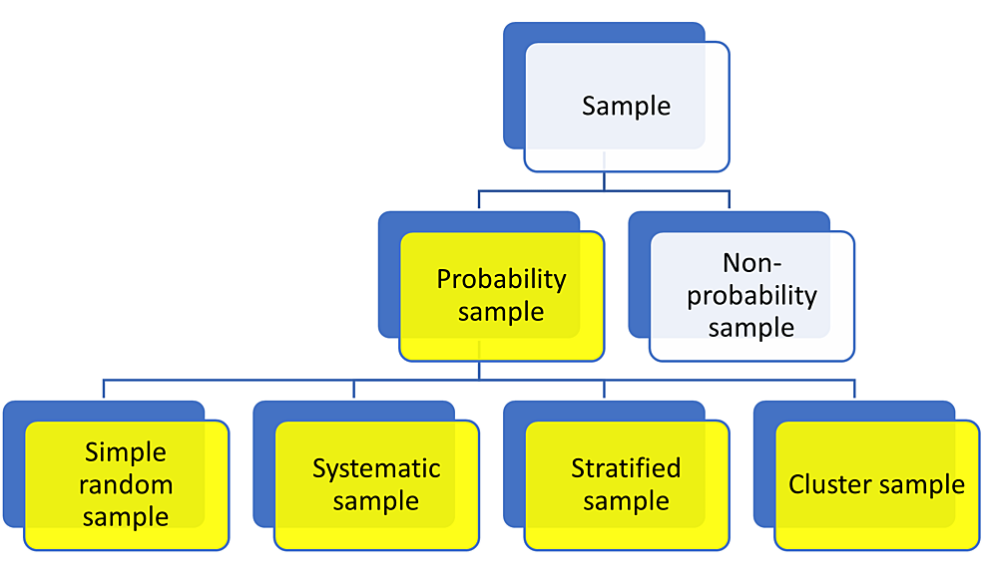
Types of sampling methods

Sampling methods are of different types and are broadly classified into probability sampling and non-probability sampling. In a probability sampling method, which is routinely employed in quantitative research, each individual in the representative population is provided with an equivalent likelihood of being included in the study [35]. Probability sampling can be separated into four types that include simple random sampling, systematic sampling, stratified sampling, and cluster sampling, as shown in Figure 6.

Simple Random Sample

In the simple random sampling method, every person in the representative population is given an equal chance of being selected. It may use a random number generator for selecting the study participants. To study the employees’ perceptions of government policies, a researcher initially assigns a number to each employee [35]. After this, the researcher randomly chooses the required number of samples. In this type of sampling method, each one has an equal chance of being selected.

Systematic Sample

In this sampling method, the researcher selects the study participants depending on a pre-defined order (1, 3, 5, 7, 9…), wherein the researcher assigns a serial number (1-100 (n)) to volunteers [35]. The researcher in this type of sample selects a number from 1 to 10 and later applies a systematic pattern to select the sample like 2, 12, 22, 32, etc.

Stratified Sample

The stratified sampling method is applied when the people from whom the sample must be taken have mixed features. In this type of sampling, the representative population is divided into clusters/strata based on attributes like age, sex, and other factors. Subsequently, a simple random or systematic sampling method is applied to select the samples from each group. Initially, different age groups, sexes, and other characters were selected as a group [35]. The investigator finds his/her sample from each group using simple or systematic random sampling methods.

Cluster Sample

This sampling method is used to create clusters of the representative population with mixed qualities. Because such groups have mixed features, each one can be regarded as a sample. Conversely, a sample can be developed by using simple random/systematic sampling approaches. The cluster sampling method is similar to stratified sampling but differs in the group characteristics, wherein each group has representatives of varied ages, different sexes, and other mixed characters [35]. Although each group appears as a sample, the researcher again applies a simple or systematic random sampling method to choose the sample.

Non-probability Sample

**Figure 7:**
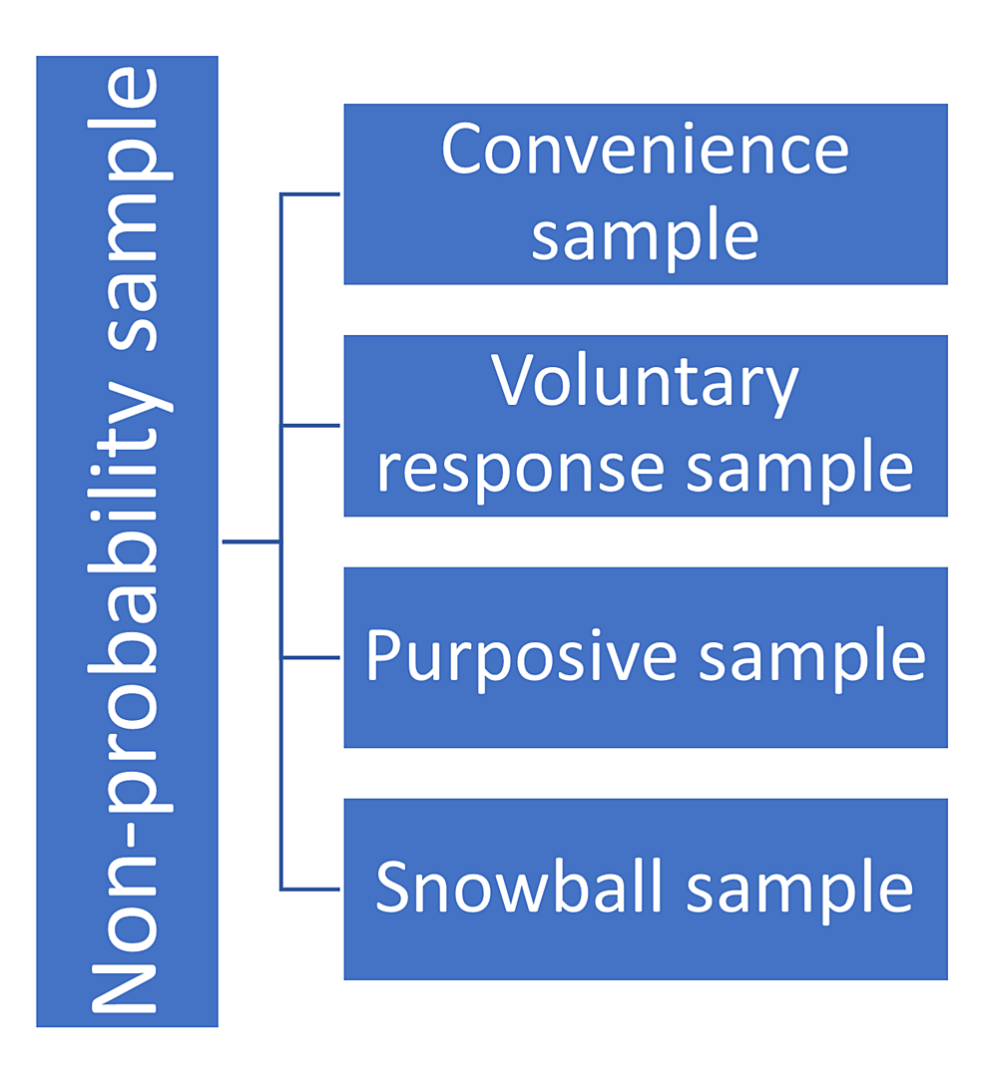
Types of non-probability sampling methods

In this type of sampling method, the participants are chosen based on non-random criteria. In a non-probability sampling method, the volunteers do not have an identical opportunity to get selected. This method, although it appears to be reasonable and effortless to do, is plagued by selection bias. The non-probability sampling method is routinely used in experimental and qualitative research. It is suitable to perform a pilot study that is carried out to comprehend the qualities of a representative population [35]. The non-probability sampling is of four types, including convenience sampling, voluntary response sampling, purposive sampling, and snowball sampling, as shown in Figure 7.

Convenience Sample

In the convenience sampling method, there are no pre-defined criteria, and only those volunteers who are readily obtainable to the investigator are included. Despite it being an inexpensive method, the results yielded from studies that apply convenience sampling may not reflect the qualities of the population, and therefore, the results cannot be generalized [35]. The best example of this type of sampling is when the researcher invites people from his/her own work area (company, school, city, etc.).

Voluntary Response Sample

In the voluntary response sampling method, the participants volunteer to partake in the study. This sampling method is similar to convenience sampling and therefore leaves sufficient room for bias [35]. The researcher waits for the participants who volunteer in the study in a voluntary response sampling method.

Purposive Sample/Judgment Sample

In the purposive or judgemental sampling method, the investigator chooses the participants based on his/her judgment/discretion. In this type of sampling method, the attributes (opinions/experiences) of the precise population group can be achieved [35]. An example of such a sampling method is the handicapped group's opinion on the facilities at an educational institute.

Snowball Sample

In the snowball sampling method, suitable study participants are found based on the recommendations and suggestions made by the participating subjects [36]. In this type, the individual/sample recruited by the investigator in turn invites/recruits other participants.

Significance of informed consent and confidentiality in health research

Informed consent is a document that confirms the fact that the study participants are recruited only after being thoroughly informed about the research process, risks, and benefits, along with other important details of the study like the time of research. The informed consent is generally drafted in the language known to the participants. The essential contents of informed consent include the aim of research in a way that is easily understood even by a layman. It must also brief the person as to what is expected from participation in the study. The informed consent contains information such as that the participant must be willing to share demographic characteristics, participate in the clinical and diagnostic procedures, and have the liberty to withdraw from the study at any time during the research. The informed consent must also have a statement that confirms the confidentiality of the participant and the protection of privacy of information and identity [37].

Health research is so complex that there may be several occasions when a researcher wants to re-visit a medical record to investigate a specific clinical condition, which also requires informed consent [38]. Awareness of biomedical research and the importance of human participation in research studies is a key element in the individual’s knowledge that may contribute to participation or otherwise in the research study [39]. In the era of information technology, the patient’s medical data are stored as electronic health records. Research that attempts to use such records is associated with ethical, legal, and social concerns [40,41]. Improved technological advances and the availability of medical devices to treat, diagnose, and prevent diseases have thrown a new challenge at healthcare professionals. Medical devices are used for interventions only after being sure of the potential benefit to the patients, and at any cost, they must never affect the health of the patient and only improve the outcome [42]. Even in such cases, the medical persons must ensure informed consent from the patients.

## Conclusions

Clinical research is an essential component of healthcare that enables physicians, patients, and governments to tackle health-related problems. Increased incidences of both communicable and non-communicable diseases warrant improved therapeutic interventions to treat, control, and manage diseases. Several illnesses do not have a treatment, and for many others, the treatment, although available, is plagued by drug-related adverse effects. For many other infections, like dengue, we require preventive vaccines. Therefore, clinical research studies must be carried out to find solutions to the existing problems. Moreover, the knowledge of clinical research, as discussed briefly in this review, is required to carry out research and enhance preparedness to counter conceivable public health emergencies in the future.
